# Supplementary material for: Quorum-sensing control of matrix protein production drives fractal wrinkling and interfacial localization of Vibrio cholerae pellicles
Source: Nat Commun. 2022 Oct 13;13:6063. doi: 10.1038/s41467-022-33816-6 (PMC9561665; doi:10.1038/s41467-022-33816-6)
Supplement: Supplementary file 1 — Supplementary Information [file 41467_2022_33816_MOESM1_ESM.pdf]

Supplementary information for:

Quorum-sensing control of matrix protein production drives fractal wrinkling and interfacial localization of *Vibrio cholerae* pellicles

Boyang Qin[1,2] and Bonnie L. Bassler[1,3]*

Affiliations:

[1]Department of Molecular Biology, Princeton University, Princeton, New Jersey 08544, USA.

[2]Department of Mechanical and Aerospace Engineering, Princeton University, Princeton, New Jersey 08544, USA.

[3]The Howard Hughes Medical Institute, Chevy Chase, Maryland 20815, USA.

*Correspondence to: B.L.B. bbassler@princeton.edu

This PDF file includes:

Supplementary figures S1-S8 and captions
Supplementary tables S1-S2

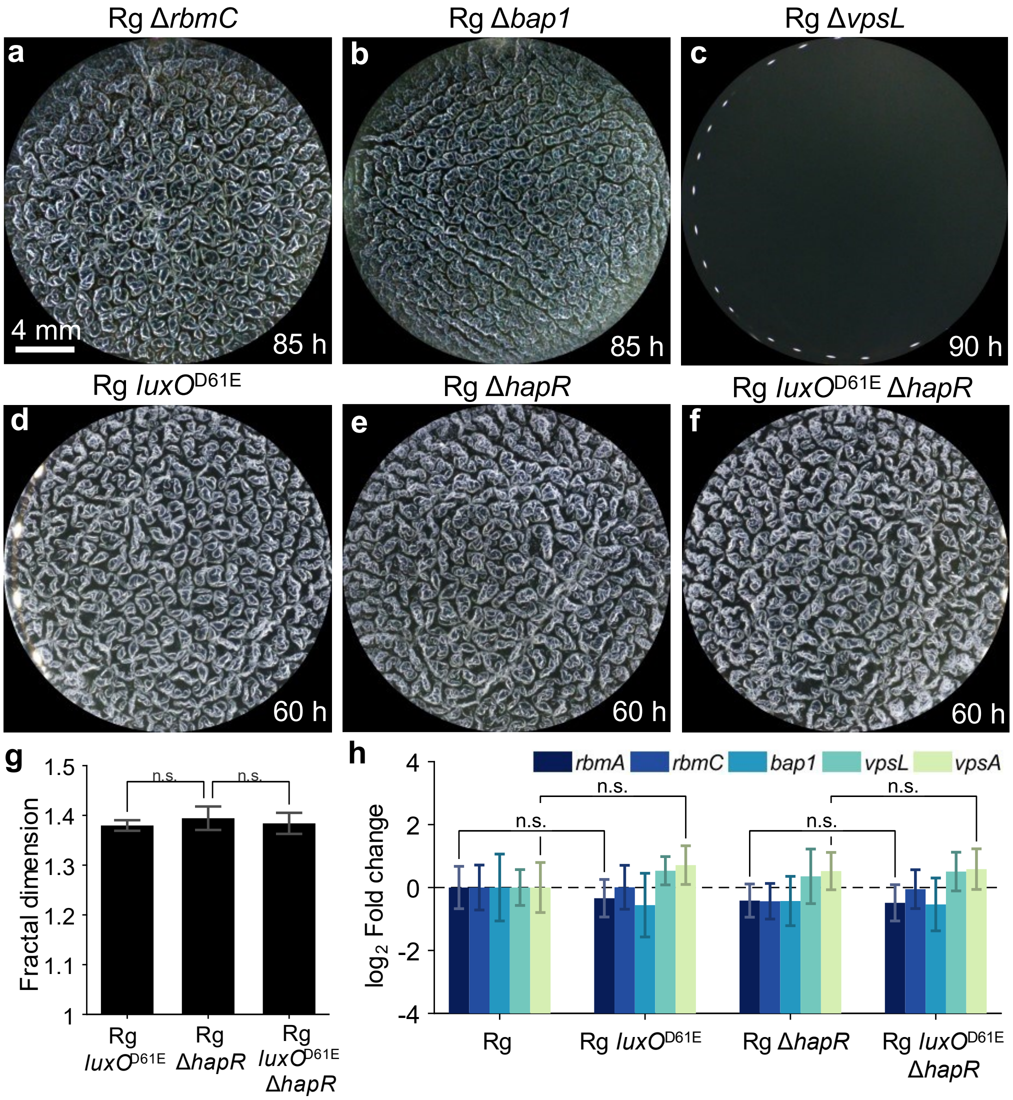

Fig. S1. ***V. cholerae*** **pellicle morphologies and corresponding matrix gene expression levels.** (**a-f**) Pellicle morphologies of the indicated strains at the indicated times. (**g**) Fractal dimensions of the wrinkle features for the indicated strains following growth for 60 h (**h**) Expression of the indicated genes in the designated strains assessed by qRT-PCR. Data are presented relative to that from the Rg strain. In (g,h), error bars denote standard deviations from biological replicates (*n* = 3). *P* values are: *, < 0.05; **, < 0.01; ***, < 0.001; ****, < 0.0001; n.s. (not significant), > 0.05; and n.d., not detected.

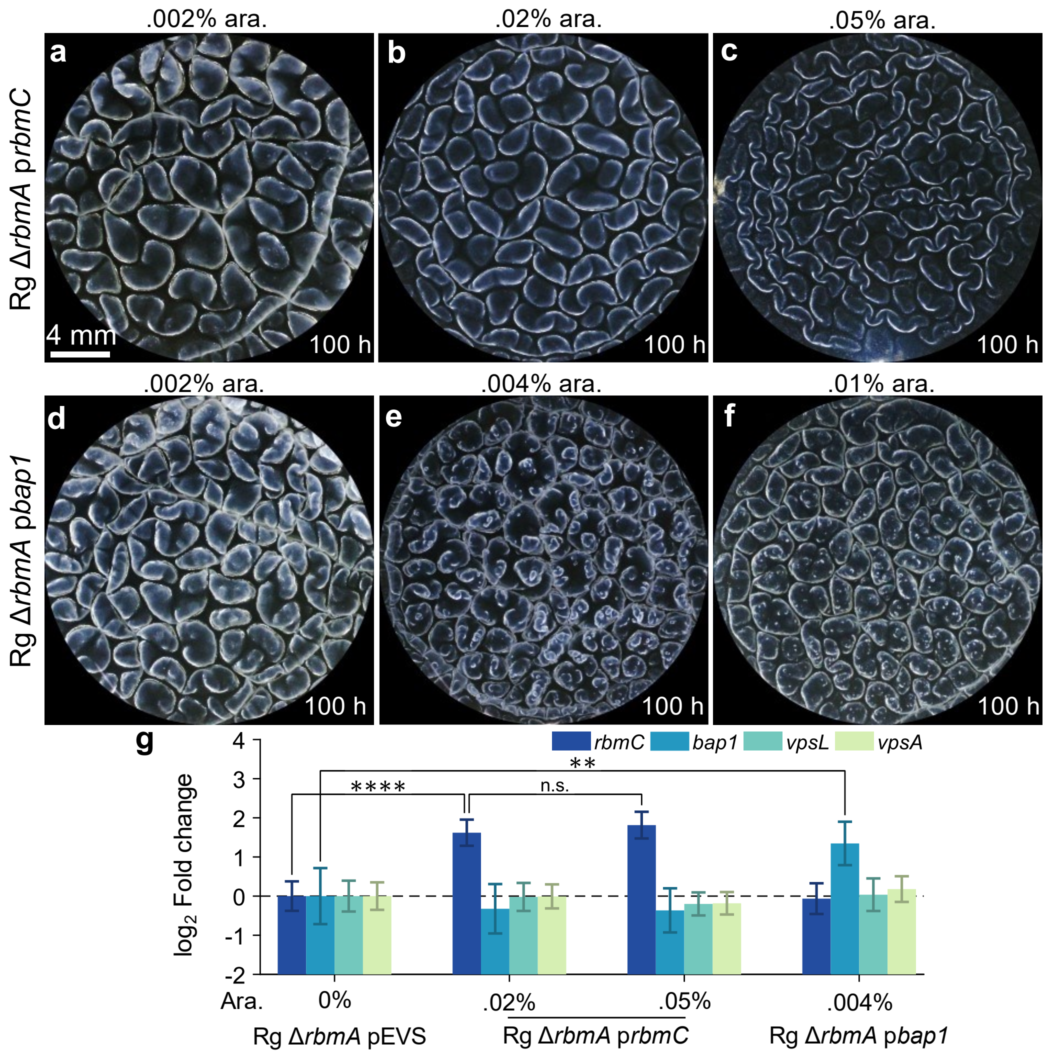

Fig. S2. Overexpression of *rbmC* or *bap1* does not rescue fractal wrinkling in the *V. cholerae* D*rbmA* mutant. (**a-f**) Pellicle morphologies for the indicated strains expressing either *rbmC* (a-c) or *bap1* (d-f) from the pEVS plasmid at the designated time points and arabinose inducer concentrations. (**g**) Expression of the indicated genes in the designated strains assessed by qRT-PCR. Data are presented relative to that from the Rg D*rbmA* strain carrying the empty pEVS vector. Error bars denote standard deviations from biological replicates (*n* = 3). *P* values are: *, < 0.05; **, < 0.01; ***, < 0.001; ****, < 0.0001; n.s. (not significant), > 0.05; and n.d., not detected.

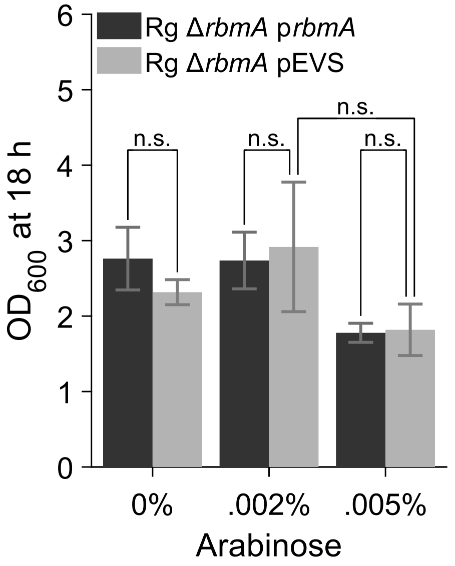

Fig. S3. Growth of strains harboring *rbmA* on pEVS and the empty pEVS vector. The OD_{600} values at 18 h are shown for the designated strains following growth in LB containing 50 μg/mL Kan at 30 ºC supplemented with arabinose at the indicated concentrations. Error bars denote standard deviations from biological replicates (*n* = 3). n.s. (not significant), $P > 0.05$.

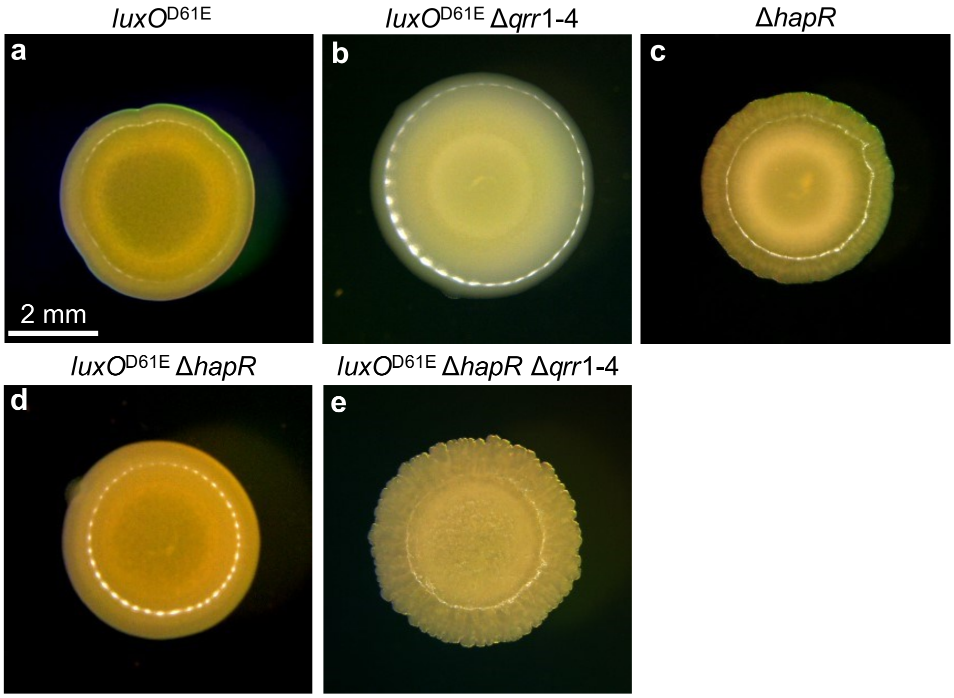

Fig. S4. Solid agar colony morphologies for QS mutants. (a-e) Colony morphologies on solid agar were imaged after 74 h of growth for the designated mutants.

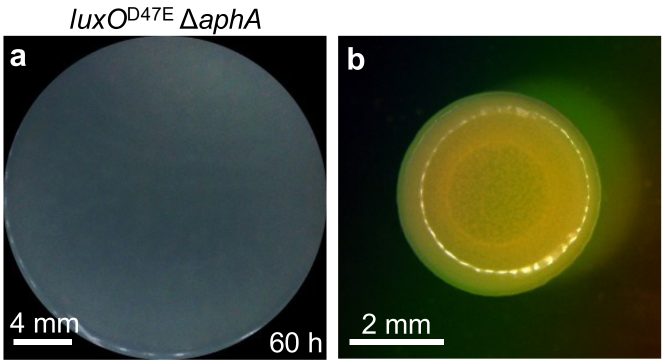

Fig. S5. AphA is required for *V. cholerae* pellicle formation. (a) Pellicle morphology of the *luxO*^{D61E} Δ*aphA* strain at 60 h. (**b**) The corresponding morphology on a solid agar surface was imaged after 51 h of growth.

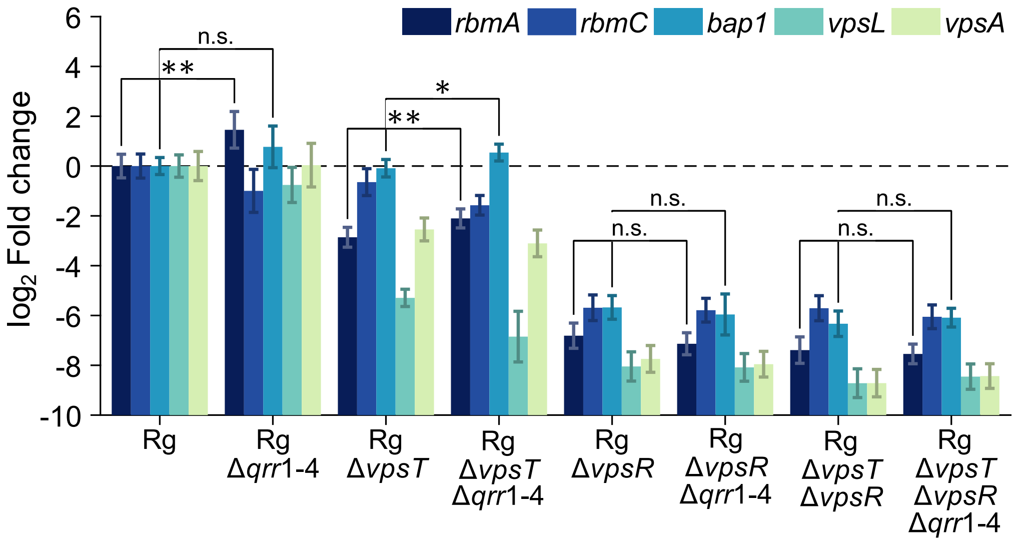

Fig. S6. The Qrr sRNAs suppress matrix gene expression via modulation of *vpsR*. Expression of the indicated genes in the designated strains assessed by qRT-PCR. Data are presented relative to that from the Rg strain. Error bars denote standard deviations from biological replicates ($n = 3$). P values are: *, < 0.05; **, < 0.01; ***, < 0.001; ****, < 0.0001; n.s. (not significant), > 0.05; and n.d., not detected.

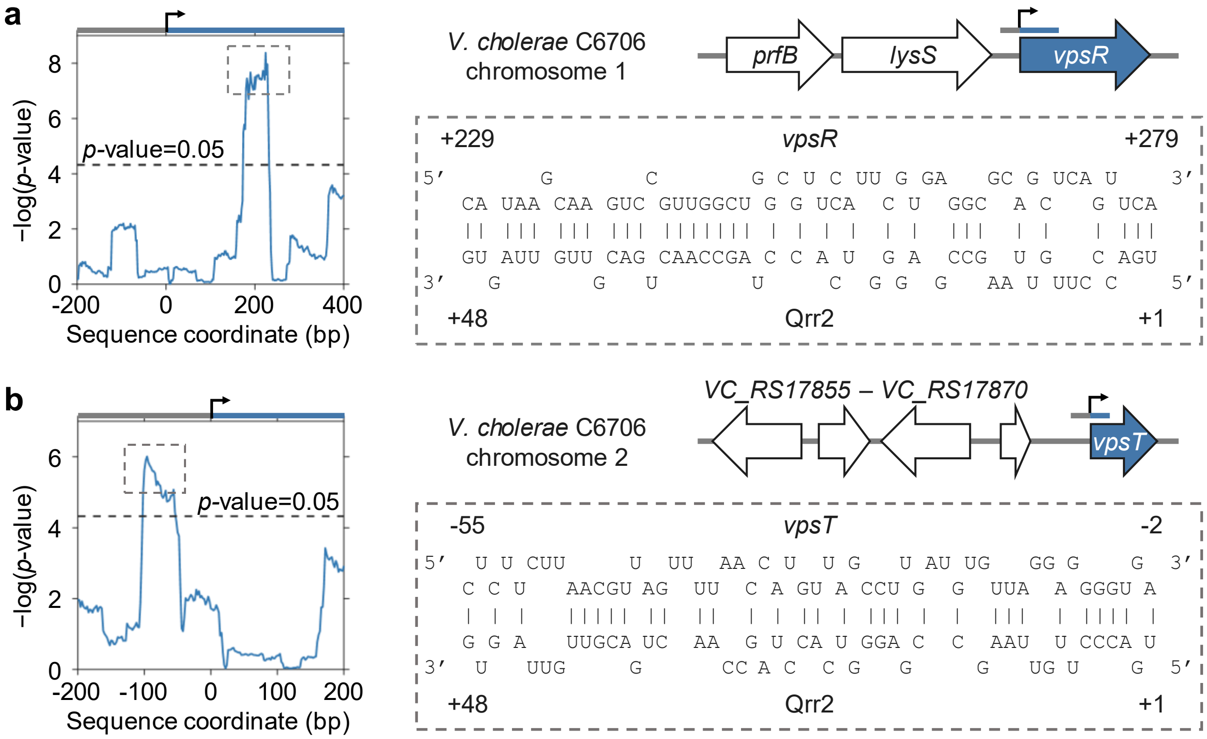

Fig. S7. Sequence analysis shows possible binding of Qrr2 to the *vpsR* and *vpsT* mRNAs. (a) Left: sequence complementarity score between Qrr2 and varying regions of the *vpsR* mRNA. The sequence coordinate is relative to the translation start site. The dashed horizontal line indicates the cutoff score. Right: putative base pairing region between Qrr2 and the *vpsR* mRNA with the highest statistical significance (indicated by the outlined box in the left panel). (**b**) Analysis as in (a) for Qrr2 and the *vpsT* mRNA.

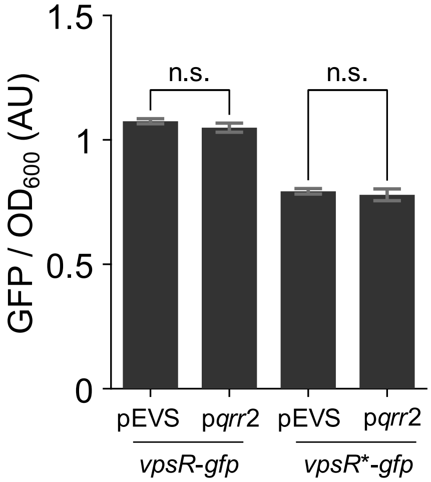

Fig. S8. Overexpression of *qrr*2 in *E. coli* does not alter *vpsR-gfp* expression. *E. coli* harboring the pEVS plasmid or pEVS with arabinose inducible *qrr*2 (p*qrr*2) were transformed with a second plasmid carrying constitutively expressed *vpsR-gfp* or *vpsR*-gfp,* in the latter, the DNA encoding the putative Qrr binding site (+223 to +279) had been randomized. Fluorescence was measured after 12 h of growth in the presence of 0.1% arabinose. Transcription of *vpsR*, *vpsR**, and *qrr*2 was verified by qRT-PCR. Error bars represent standard deviations of three replicates ($n = 3$). n.s. denotes not significant ($P > 0.05$).

Table S1. Strains and plasmids used in this study.

Strain/Plasmid	Relevant features	Source
V. cholerae		
C6706str2	O1 El Tor wild type	[1]
BH038	*luxO*^{D61E}	[2]
MM194	Δ*hapR*	[3]
BQ145A	Δ*hapR luxO*^{D61E}	This study
MJ260	*luxO*^{D61E} Δ*aphA*	Bassler lab collection
SLS641	*luxO*^{D61E} Δ*hapR* Δ*qrr*1-4	Bassler lab collection
SLS643	*luxO*^{D61E} Δ*qrr*1-4	Bassler lab collection
BQ153A	*luxO*^{D61E} pCN013	This study
BQ154A	*luxO*^{D61E} pEVS143	This study
BQ165A	*luxO*^{D61E} Δ*hapR* Δ*qrr*1-4, Δ*vpsR*	This study
BQ166A	*luxO*^{D61E} Δ*hapR* Δ*qrr*1-4, Δ*vpsT*	This study
BQ167A	*luxO*^{D61E} Δ*hapR* Δ*qrr*1-4 Δ*vpsT* Δ*vpsR*	This study
BQ168A	*luxO*^{D61E} Δ*hapR* Δ*vpsR*	This study
BQ169A	*luxO*^{D61E} Δ*hapR* Δ*vpsT*	This study
BQ170A	*luxO*^{D61E} Δ*hapR* Δ*vpsT* Δ*vpsR*	This study
BQ178A	*luxO*^{D61E} Δ*hapR* Δ*qrr*1-4 pBQ034	This study
BQ195A	*luxO*^{D61E} Δ*hapR* Δ*qrr*1-4 pEVS143	This study
JY028	*vpvC*^{W240R}	[4]
JY038	*vpvC*^{W240R} Δ*vpsL*	[4]
JY046	*vpvC*^{W240R} Δ*rbmA*	[4]
JY301	*vpvC*^{W240R} Δ*rbmC* Δ*bap1*	[4]
JY312	*vpvC*^{W240R} Δ*rbmA* Δ*rbmC* Δ*bap1*	[4]
JY473	*vpvC*^{W240R} Δ*rbmA* Δ*rbmC* Δ*bap1* Δ*vpsL* Δ*Vc1807*::*SpecR*	Bassler lab collection
JY291	*vpvC*^{W240R} *luxO*^{D61E}	Bassler lab collection
BQ221A	*vpvC*^{W240R} Δ*rbmA* pCN013	This study
BQ223A	*vpvC*^{W240R} Δ*hapR*	This study
BQ227A	*vpvC*^{W240R} Δ*rbmA* pEVS143	This study
BQ231A	*vpvC*^{W240R} *luxO*^{D61E} Δ*hapR*	This study
BQ239A	*vpvC*^{W240R} Δ*rbmA* pCN014	This study
BQ253A	*vpvC*^{W240R} Δ*rbmA* pBQ036	This study
BQ242A	*vpvC*^{W240R} Δ*vpsR*	This study
BQ243A	*vpvC*^{W240R} Δ*vpsT*	This study
BQ244A	*vpvC*^{W240R} Δ*vpsR* Δ*vpsT*	This study
BQ245A	*vpvC*^{W240R} Δ*qrr*1-4	This study
BQ246A	*vpvC*^{W240R} Δ*qrr*1-4 Δ*vpsR*	This study
BQ247A	*vpvC*^{W240R} Δ*qrr*1-4 Δ*vpsT*	This study

BQ248A	*vpvC*^{W240R} Δ*qrr*1-4 Δ*vpsR* Δ*vpsT*	This study
E. coli		
BQEc80	pTetO-*vpsR*-*gfp,* pEVS143	This study
BQEc81	pTetO-*vpsR*-*gfp,* pEVS143 *araC*-pBAD-*qrr*2	This study
BQEc82	pTetO-*vpsR*-gfp,* pEVS143	This study
BQEc83	pTetO-*vpsR*-gfp,* pEVS143 *araC*-pBAD-*qrr*2	This study
Plasmids		
pKAS32	Suicide vector, Amp^{R} Sm^{S}	[5]
pEVS143	pEVS143 empty backbone, Kan^{R}	[6]
pNUT144	Suicide vector, Amp^{R} Kan^{R} Sm^{S}	[7]
pNUT157	pNUT144 *vpvC*^{W240R}	[7]
BBEc0591	pKAS32 Δ*hapR*	[8]
pCN013	pEVS143 *araC*-pBAD-*rbmA*	[7]
pCN014	pEVS143 *araC*-pBAD-*rbmC*	[7]
pBH2	pKAS32 Δ*vpsR*	[2]
JC480	pKAS32 Δ*vpsT*	Bassler lab collection
pBQ034	pEVS143 *araC*-pBAD-*qrr*2	This study
pBQ036	pEVS143 *araC*-pBAD-*bap1*	This study
pBQ076	pTetO-*vpsR*-*gfp*	This study
pBQ078	pTetO-*vpsR*-gfp*	This study

Table S2. Oligonucleotides used in the study.

Index	Name	Sequence	Purpose
BQ128	VC1258_gyrA_RT_F	TGGCCAGCCAGAGATCAAG	qRT-PCR: *gyr*
BQ129	VC1258_gyrA_RT_R	ACCCGCAGCGGTACGAC	qRT-PCR: *gyr*
BQ130	VC0928_rbmA_RT_F	GTTGATACAGCGCAGCAAGAGT	qRT-PCR: *rbmA*
BQ131	VC0928_rbmA_RT_R	CACGTGTAACGTTCAACATACG	qRT-PCR: *rbmA*
BQ132	VC0930_rbmC_RT_F	CAGGCTATGCCGCTGTCGATAT	qRT-PCR: *rbmC*
BQ133	VC0930_rbmC_RT_R	TGCCAGTGTCGTCATTCGCCAC	qRT-PCR: *rbmC*
BQ134	VC0917_vpsA_RT_F	TGAGTAGTGAACAGCCAGATGTG	qRT-PCR: *vpsA*
BQ135	VC0917_vpsA_RT_R	CGGAATTTGTTGATAATACGCGGC	qRT-PCR: *vpsA*
BQ138	VC1888_bap1_RT_F	AGTGAAATCGCTGTGCTTGAGC	qRT-PCR: *bap1*
BQ139	VC1888_bap1_RT_R	GCTTGTACGCTACCACCTG	qRT-PCR: *bap1*
BQ140	VCA0952_vpsT_RT_F	AGTCGCAGTATTCAGATGCTG	qRT-PCR: *vpsT*
BQ141	VCA0952_vspT_RT_R	TCGCATCAGGACAACTGATGTG	qRT-PCR: *vpsT*
BQ142	VC0934_vpsL_RT_F	CAGTATGCGAGTGATGGATAATGG	qRT-PCR: *vpsL*
BQ143	VC0934_vpsL_RT_R	TCGTGGATCGCCTTTGGT	qRT-PCR: *vpsL*
BQ148	Vc_qrr_RT_F1	CACCTAGCCAACTGACGTTGT	qRT-PCR: *qrr*1-4
BQ149	Vc_qrr1_RT_R1	TGAGTCTATTGGCTGTTATTTGTGA	qRT-PCR: *qrr*1
BQ150	Vc_qrr2_RT_R1	CGCGATTGGCTTATATATGATGT	qRT-PCR: *qrr*2
BQ151	Vc_qrr3_RT_R1	CAAAAGGGTGATTGGCTGATA	qRT-PCR: *qrr*3
BQ152	Vc_qrr4_RT_R1	AGAAGTGTGATTGGCCGTCT	qRT-PCR: *qrr*4
BQ170	Vc_hapR_RT_F3	ACCGAACTAACCAACTGCTGAT	qRT-PCR: *hapR*
BQ171	Vc_hapR_RT_R3	AACAGGCTGGCCATGTTATC	qRT-PCR: *hapR*
BQ178	Vc_aphA_RT_F3	AAGCACAACAACCGTTTAGATAGA	qRT-PCR: *aphA*
BQ179	Vc_aphA_RT_R3	CTTTCCAGAAGTAGCCAATGC	qRT-PCR: *aphA*
BQ256	VC0665_vpsR_RT_F	GTCAGTATTTTGATCTATGGCGAAAGTGG	qRT-PCR: *vpsR*
BQ257	VC0665_vpsR_RT_R	GGCAATTAACTGAAATGAAAGGTTTTTGTGC	qRT-PCR: *vpsR*
BQ220	pEVS143_extract_dns_F	GATCCGGTGATTGATTGAGCAAGC	pEVS143 plasmid integration
BQ221	pEVS143_extract_RBS_R	TTTAGACCTCCTGCGGCCGC	pEVS143 plasmid integration
BQ222	qrr2_ext_pEVS143_RBS_F	GCGGCCGCAGGAGGTCTAAATGACCCTTGT TAAGCCGAGGG	*qrr*2 integration to pEVS143
BQ223	qrr2_ext_pEVS143_dns_R	GCTTGCTCAATCAATCACCGGATCCTAATTA TCAACAAAAAAGCAGTGAAAATAGCGGG	*qrr*2 integration to pEVS143
BQ266	bap1_ext_pEVS143_pBAD _F	GCGGCCGCAGGAGGTCTAAAATGAAACAGA CAAAAACGTTGACCG	*bap1* integration to pEVS143
BQ267	bap1_ext_pEVS143_pBAD _R	GCTTGCTCAATCAATCACCGGATCCAGAAG TATCTTTACTTCAGCGGAACGC	*bap1* integration to pEVS143
BQ379	VC0665_vpsR_RT_F2	GATTCAGTACCTGGCTCTCTTGTTGTG	qRT-PCR: *vpsR*
BQ380	VC0665_vpsR_RT_R2	CTTTGCGCAAATCTGCTACTTGAGTAC	qRT-PCR: *vpsR*
BQ381	Ec_cysG_RT_F	CGTTTATTCCACAGTTCACCGCATG	qRT-PCR: *cysG*
BQ382	Ec_cysG_RT_R	GTTAAGCGCGTCATCATCCGTC	qRT-PCR: *cysG*

References

1. Thelin, K. H. & Taylor, R. K. Toxin-coregulated pilus, but not mannose-sensitive hemagglutinin, is required for colonization by *Vibrio cholerae* O1 El Tor biotype and O139 strains. *Infect Immun.* **64**, 2853–2856 (1996).
2. Hammer, B. K. & Bassler, B. L. Quorum sensing controls biofilm formation in *Vibrio cholerae*. *Mol. Microbiol.* **50**, 101–104 (2003).
3. Miller, M. B., Skorupski, K., Lenz, D. H., Taylor, R. K. & Bassler, B. L. Parallel quorum sensing systems converge to regulate virulence in *Vibrio cholerae*. *Cell* **110**, 303–314 (2002).
4. Yan, J., Sharo, A. G., Stone, H. A., Wingreen, N. S. & Bassler, B. L. *Vibrio cholerae* biofilm growth program and architecture revealed by single-cell live imaging. *Proc. Natl. Acad. Sci. U.S.A.* **113**, E5337 (2016).
5. Skorupski, K. & Taylor, R. K. Positive selection vectors for allelic exchange. *Gene* **169**, 47–52 (1996).
6. Dunn, A. K., Millikan, D. S., Adin, D. M., Bose, J. L. & Stabb, E. V. New rfp- and pES213-derived tools for analyzing symbiotic *Vibrio fischeri* reveal patterns of infection and *lux* expression in situ. *Appl. Environ. Microbiol.* (2006) doi:10.1128/AEM.72.1.802-810.2006.
7. Drescher, K., Nadell, C. D., Stone, H. A., Wingreen, N. S. & Bassler, B. L. Solutions to the public goods dilemma in bacterial biofilms. *Curr. Biol.* **24**, 50–55 (2014).
8. Zhu, J. *et al.* Quorum-sensing regulators control virulence gene expression in *Vibrio cholerae*. *Proc. Natl. Acad. Sci. U.S.A.* **99**, 3129–3134 (2002).
