# Supplementary material for: Quorum-sensing control of matrix protein production drives fractal wrinkling and interfacial localization of Vibrio cholerae pellicles
Source: Nat Commun. 2022 Oct 13;13:6063. doi: 10.1038/s41467-022-33816-6 (PMC9561665; doi:10.1038/s41467-022-33816-6)
Supplement: Supplementary file 3 — Description of Additional Supplementary Files [file 41467_2022_33816_MOESM3_ESM.pdf]

Description of Additional Supplementary Files:

Movie S1. Top-view time lapse images of pellicle morphogenesis by the *V. cholerae* Rg Δ*rbmA* strain at a liquid-liquid interface.

Movie S2. Top-view time lapse images of pellicle morphogenesis by the *V. cholerae* Rg Δ*rbmC* Δ*bap1* strain.

Movie S3. Side-view time lapse images of pellicle morphogenesis by the *V. cholerae* Rg Δ*rbmC* Δ*bap1* strain.

Movie S4. Time lapse images of the top-view focus projection of pellicle morphogenesis by the *V. cholerae* Rg Δ*rbmA* p*rbmA* strain induced with 0.002% arabinose.

Movie S5. Top-view time lapse images of pellicle morphogenesis by the *V. cholerae* $luxO^{D61E}$ strain.

Movie S6. Top-view time lapse images of pellicle morphogenesis by the *V. cholerae* Δ*hapR* strain.

Movie S7. Top-view time lapse images of pellicle morphogenesis by the *V. cholerae* $luxO^{D61E}$ Δ*hapR* strain.

Movie S8. Top-view time lapse images of pellicle morphogenesis by the *V. cholerae* $luxO^{D61E}$ Δ*hapR* Δ*qrr*1-4 strain.

Movie S9. Top-view time lapse images of pellicle morphogenesis by the *V. cholerae* $luxO^{D61E}$ Δ*hapR* Δ*qrr*1-4 p*qrr*2 strain induced with 0.01% arabinose.
